# Magnetic resonance imaging for the non-invasive diagnosis in patients with ovarian cancer: A protocol for systematic review and meta-analysis

**DOI:** 10.1097/MD.0000000000023551

**Published:** 2020-12-11

**Authors:** Yongxue Su, Lingli Deng, Lijun Yang, Xianhong Yuan, Wei Xia, Ping Liu

**Affiliations:** a Department of Radiology; b Department of Obstetrics, Maternal and Child Health Hospital of Hubei Province; c Department of Imaging Center, Wuhan Children's Hospital (Wuhan Maternal and Child Healthcare Hospital), Tongji Medical College, Huazhong University of Science and Technology, Wuhan, Hubei, P. R. China.

**Keywords:** magnetic resonance imaging, non-invasive diagnosis, ovarian cancer, systematic review

## Abstract

**Background:**

In developed nations, ovarian cancer has resulted in the most fatalities from gynecological cancer. Laparoscopy is primarily utilized as the test to diagnose ovarian cancer. Besides being costly, there are surgical risks associated with laparoscopies. At present, clinical practitioners have access to non-invasive tests for diagnosing ovarian cancer. This study aims to evaluate the diagnostic accuracy of magnetic resonance imaging (MRI) for diagnosing ovarian cancer.

**Methods:**

In order to obtain eligible studies, cross-sectional studies or randomized controlled trials are searched in electronic databases. The databases include 5 English databases (PubMed, the Cochrane Library, PsycINFO, EMBASE, and Web of Science) and 3 Chinese databases (China Biomedical Literature Database, China National Knowledge Infrastructure, and WanFang database). The databases are searched from their origin to October 2020. Quality Assessment of Diagnostic Accuracy Studies-2 is used to assess the methodological quality of the selected studies. RevMan 5.3 and SAS NLMIXED software are used to assess the data synthesis, sensitivity analysis, and risk of bias assessment.

**Results:**

This study evaluates the pooled diagnostic value of MRI for diagnosing ovarian cancer.

**Conclusions:**

This study will summarize previously published evidence of MRI in relation to diagnosing ovarian cancer.

**Ethics and dissemination:**

Since this study does not utilize data from patients, this protocol does not require ethical approval.

**Protocol registration number:**

DOI 10.17605/OSF.IO/A6SPQ (https://osf.io/a6spq)

## 1 Introduction

Ovarian cancer is a deadly form of cancer. It causes the most fatalities from gynecological carcinoma. In 2020, there has been approximately 21,750 new cases of cancer in the United States with 13,940 fatalities.^[1,2]^ The combination of cytoreductive surgery with complete resection of all macroscopic disease and platinum-based chemotherapy is the existing standard of treatment for ovarian cancer.^[3–5]^ Ovarian cancer is primarily diagnosed via a laparoscopy. However, it is expensive and there is a probability of surgical risks. As a result, imaging tests have been considered for their potential in the diagnosis of ovarian cancer in a non-invasive way. An accurate imaging test has the potential to replace surgery in the diagnosis of ovarian cancer, or in the very least, it can minimize the need for surgery. Importantly, if imaging tests can precisely pinpoint the location of ovarian cancer, surgeons can use the information to gain an upper hand when planning the surgery, this improves the chances of a positive outcome.

Over the years, magnetic resonance imaging (MRI) has shown increasing potential in diagnosing ovarian cancer.^[6–9]^ However, there has been no systematic review on the diagnostic accuracy of MRI when detecting ovarian cancer non-invasively. Therefore, this study is conducted to evaluate the accuracy of MRI for diagnosing ovarian cancer. It aims to collect reliable evidence to provide clinical guidance and to help ovarian cancer patients to seek more reasonable diagnostic methods. To this end, this study will evaluate the diagnostic accuracy of MRI in diagnosing ovarian cancer.

## 2 Methods

### 2.1 Study registration

This protocol has been registered on Open Science Framework (OSF, http://osf.io/) with the registration DOI number 10.17605/OSF.IO/A6SPQ. It is reported in accordance with the guideline of the Preferred Reporting Items for Systematic Reviews and Meta-Analysis Protocol (PRISMA-P) Statement.^[10]^

### 2.2 Inclusion criteria for study selection

#### 2.2.1 Type of studies

This study will include cross-sectional studies or randomized controlled trials that has evaluated the diagnostic accuracy of MRI for the diagnosis of ovarian cancer.

#### 2.2.2 Type of participants

Participants include adults (aged over 18 years), females with suspected ovarian cancer based on clinical symptoms and/or pelvic examination.

#### 2.2.3 Type of index test

##### 2.2.3.1 Index test

MRI was utilized in women with ovarian cancer.

##### 2.2.3.2 Reference standards

The reference standard included visualization of ovarian cancer surgery with or without histological confirmation.

#### 2.2.4 Types of outcome measures

Types of outcome measures include sensitivity, specificity, positive likelihood ratio, negative likelihood ratio, diagnosis odds ratio, and area under the curve.

### 2.3 Information sources and search strategy

Cross-sectional studies or randomized controlled trials will be searched for potential eligible studies. The search will be performed in electronic databases, these include 5 English databases (PubMed, the Cochrane Library, PsycINFO, EMBASE, and Web of Science) and 3 Chinese databases (China Biomedical Literature Database, China National Knowledge Infrastructure, and WanFang database). The databases will be searched from their origin till October 2020. Furthermore, additional sources will also be examined, such as Google Scholar (https://scholar.google.com.hk/) and ClinicalTrials.gov (https://clinicaltrials.gov/). The reference lists of all relevant studies and grey literature will also be included in the search to prevent missing any potential article. The following MeSH terms, related synonym, and their combinations will be used to search the databases mentioned before: “ovarian neoplasm,” “ovarian cancer,” “ovarian tumor,” “ovarian carcinoma,” “magnetic resonance imaging,” MRI,∗ “cross-sectional study,” “cross-sectional study,∗” “randomized controlled trial,” “randomised controlled trial,” randomly,∗ and randomised controlled trials.∗

### 2.4 Information sources and search strategy

#### 2.4.1 Study selection

**Figure 1:**
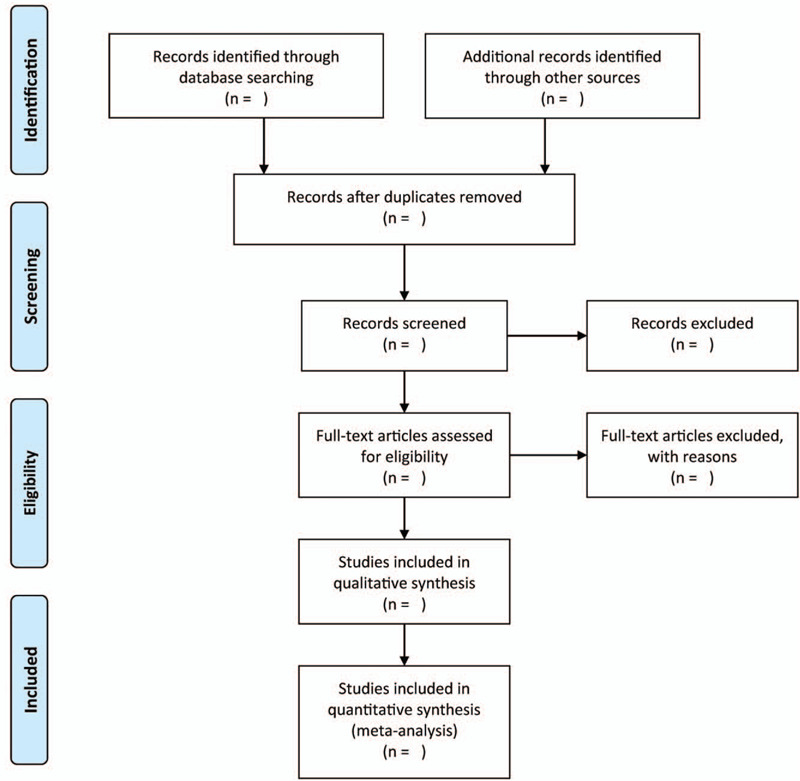
The research flowchart.

EndNote X9 is used to manage all search records. It is also used to remove duplicated publications. The titles and abstracts will be filtrated independently by 2 authors. Once potential articles are selected, the full text will be appraised for further selection based on the inclusion and exclusion criteria. All disagreements will be documented and resolved through discussion, or when necessary by consulting a third author. Figure 1 illustrates the selection flow chart.

#### 2.4.2 Data extraction and management

The data will be extracted independently by 2 authors using a pre-designed form. In the case of missing information or ambiguousness, the authors of the study will be contacted in an attempt to obtain the missing information or clear any doubts. All disagreements are documented and will be resolved through discussion or by consulting a third author where necessary. The data extracted from the studies include the following: author, publication date, study design, and sample size, age, gender, ethnicity, tumor stage, pathologic tumor size, sensitivity, specificity, false negatives, false positives, index test and reference standard, and other critical information.

#### 2.4.3 Assessment of methodological quality

Quality Assessment of Diagnostic Accuracy Studies-2 is used to assess the methodological quality of the selected studies.^[11]^

#### 2.4.4 Measures of treatment effect

The sensitivity, specificity, positive likelihood ratio, negative likelihood ratio, diagnosis odds ratio, and area under the curve will be calculated using the numbers of true and false negatives and positives.

#### 2.4.5 Assessment of heterogeneity

The heterogeneity between studies will be assessed using the I2 value. It is considered that *I*^2^ > 50% indicates significant heterogeneity, in this case, the random-effects model will be used to merge data.^[12]^ However, if the fixed-effects model will be used to merge data.^[13]^

#### 2.4.6 Sensitivity analysis

If sufficient data is available a sensitivity analysis will be conducted to detect the stability of our findings.

#### 2.4.7 Assessment of reporting biases

Funnel plots will be used to evaluate the potential publication bias if there is any form of reporting bias.

## 3 Discussion

To the best knowledge of the author, there has been no previously published systematic review pertaining to the diagnostic accuracy of MRI when detecting ovarian cancer non-invasively. The present systematic review and meta-analysis is the first to evaluate the diagnostic accuracy of MRI for the diagnosis of ovarian cancer. This study will summarize current published evidence to provide direct and indirect evidence and provide future direction for studies focused on ovarian cancer diagnosis.

## Author contributions

**Conceptualization:** Yongxue Su, Ping Liu.

**Data curation:** Yongxue Su, Lingli Deng, Wei Xia, Ping Liu.

**Formal analysis:** Lingli Deng, Xianhong Yuan, Ping Liu.

**Funding acquisition:** Lijun Yang, Xianhong Yuan.

**Investigation:** Lingli Deng, Lijun Yang, Ping Liu.

**Methodology:** Lingli Deng, Lijun Yang.

**Project administration:** Wei Xia.

**Resources:** Lingli Deng, Lijun Yang, Wei Xia.

**Software:** Yongxue Su, Xianhong Yuan, Ping Liu.

**Supervision:** Lijun Yang, Wei Xia.

**Validation:** Lijun Yang, Xianhong Yuan, Ping Liu.

**Visualization:** Ping Liu.

**Writing – original draft:** Yongxue Su, Ping Liu.

**Writing – review & editing:** Yongxue Su, Ping Liu.
